# Model-Based Characterization of the Metabolism of Recombinant Adeno-Associated Virus (rAAV) Production via Human Embryonic Kidney (HEK293) Cells

**DOI:** 10.3390/bioengineering12040345

**Published:** 2025-03-27

**Authors:** Somaiyeh Khodadadi Karimvand, Miroslava Cuperlovic-Culf, Amine A. Kamen, Miodrag Bolic

**Affiliations:** 1 School of Electrical Engineering and Computer Science, University of Ottawa, Ottawa, ON K1N 6N5, Canada; s.khodadadi.chem85@gmail.com; 2 Digital Technologies Research Center, National Research Council, Ottawa, ON K1A 0R6, Canada; miroslava.cuperlovic-culf@nrc-cnrc.gc.ca; 3 Department of Biochemistry, Microbiology, and Immunology, Faculty of Medicine, University of Ottawa, Ottawa, ON K1H 8M5, Canada; 4 Department of Bioengineering, McGill University, Montreal, QC H3A 0E9, Canada; amine.kamen@mcgill.ca

**Keywords:** adeno-associated viruses, Michaelis–Menten kinetics, metabolic model, global sensitivity analysis, optimization, model fitting

## Abstract

In this paper, we present a kinetic–metabolic model describing adeno-associated virus (AAV) production via HEK293 cells that encompasses the main metabolic pathways, namely, glycolysis, tricarboxylic acid cycle (TCA), pyruvate fates, the pentose phosphate pathway, anaplerotic reaction, amino acid metabolism, nucleotides synthesis, biomass synthesis, and the metabolic pathways of protein synthesis of the AAV (capsid and Rep proteins). For the modeling, Michaelis–Menten kinetics was assumed to define the metabolic model. A dataset from bioreactor cultures containing metabolite profiles of adeno-associated virus 6 (AAV6) production via triple transient transfection in a low-cell-density culture, including the concentration profiles of glutamine, glutamic acid, glucose, lactate, and ammonium, was utilized for fitting and computing the model parameters. The model that resulted from the adjusted parameters defined the experimental data well. Subsequently, a Sobol-based global sensitivity analysis procedure was applied to determine the most sensitive parameters in the final model.

## 1. Introduction

Adeno-associated viruses (AAVs), which belong to the Parvoviridae family, are small single-stranded DNA viruses that are non-enveloped and inherently unable to replicate independently. Therefore, they need a helper virus (adenovirus or herpes simplex virus (HSV)) to provide the genes required for vector replication. The AAV is characterized by a small protein capsid that contains a single-stranded DNA genome. Due to their inability to generate disease, AAVs are excellent candidates for gene therapy [1]. Their high safety due to low toxicity, robust and long-term transgene expression, and the availability of viral serotypes have recently made recombinant adeno-associated virus (rAAV) vectors some of the most widely used outstanding choices for gene delivery vectors in the pre-clinical models of human disease. The increase in demand for gene therapy products has created the need to produce large quantities of rAAVs.

A mammalian cell culture platform, e.g., Chinese hamster ovary (CHO) cells and Human Embryonic Kidney 293 (HEK 293), has been successfully employed in the technology of industrial biopharmaceutical drugs through the advancements in their capability to express human proteins with proper processing. However, due to the lack of certain sugar-transferring enzymes, CHO cells cannot perform all types of human glycosylation [2]. Furthermore, by producing immunogenic glycan structures, CHO cells can increase the drug’s clearance and reduce the efficacy [3]. 

CHO cells naturally release virus-like particles (VLPs) into the culture medium. These VLPs must either be inactivated or eliminated from the final recombinant product during downstream processing (DSP). Thus, another significant limitation of CHO cells lies in their tendency to produce retroviral VLPs, which cannot be separated from AAV particles [4].

The most common cell lines used in the production of AAVs for preclinical and clinical studies are insect cells, mainly Spodoptera frugiperda (Sf9) cells, Henrietta Lacks (HeLa) cells, Baby Hamster Kidney (BHK) cells [5], and the transient transfection of HEK293 cells. Currently, HEK293 is known as the most extensively used human cell line for the transient transfection of rapid production of rAAVs. HEK293 cells are the preferred cell expression platform for rAAV production. Several metabolic network models have been reported in the literature for the HEK293 cell line. Abbate et al. recently presented a detailed model of HEK293 metabolism [6]. It should be noted that changes in the cell metabolism of HEK293 cells due to the transfection effect can change the rAAV viral vector production process.

Today, there is an urgent need to improve rAAV manufacturing to fulfill the high requirements of the viral vectors for preclinical/clinical studies. However, the transfection-based manufacturing of rAAV viral vectors remains challenging due to low yields of final production and a low fraction of full capsids in the harvesting step [7,8]. In other words, the main problem of rAAV production is the low yield of capsids containing a therapeutic element, which leads to high costs due to additional purification steps to remove empty particles. Consequently, increasing attention has been directed to optimizing the vector titer, purification of high-titer rAAVs, and increasing the productivity [4]. Thus, different strategies, such as chromatography modes, including size exclusion and ion exchange, were developed for AAV purification [9,10]. The increasing use of AAVs as a vector system is mostly due to the very efficient long-term expression of the therapeutic gene in terminally differentiated cells and the simplicity of the genome. Since an AAV requires a helper virus to replicate successfully in human cells, a significant change in the development of AAV design was the use of plasmids for rAAVs production, and the most common technique is the utilization of the triple transfection of HEK293 cells. Nguyen et al. 2021 presented a mechanistic model for synthesizing rAAV viral vectors by triple plasmid transfection in the biomanufacturing process [11].

Metabolic modeling is a powerful tool emerging as a promising alternative to the study, design, and quantitative evaluation of biological processes. Achieving such models requires the use of kinetic models to encapsulate their behavior. Indeed, mathematical kinetic modeling provides the optimal framework for characterizing the dynamic properties of mammalian cell culture [12,13,14].

Nowadays, developing in silico mathematical models to understand the mechanism of processes; predict the responses to various process inputs; and thus, minimize the time and cost of production optimization can be considered a promising tool. The mechanical models, developed based on chemical and biological principles, can help us understand the mechanisms and regulation of cell growth and production. However, due to the biological process’s complexity and our lack of knowledge, it is usually difficult to develop a completely accurate model.

To our knowledge, a detailed kinetic mechanistic model related to AAV production in the HEK293 cell line platform has not been reported yet. This study aimed to present a mechanical, mathematical model for the metabolic pathways of the rAAV viral vector production process within the HEK293 cell platform based on flux kinetic expressions. Indeed, a metabolic model framework based on flux kinetics was developed. The model was successfully validated by an experimental metabolic dataset of a bioreactor culture to optimize the model parameters. In what follows, the sensitivity analysis of the optimized parameters is performed to evaluate their impacts on the final quantity of production progress.

## 2. Theoretical Background

All data analysis tasks, including the model fitting and sensitivity analysis, were conducted using the MATLAB 2019b software environment.

### 2.1. Fitting Process

To obtain precise parameter constants, the kinetic process was fitted using the efficient, robust, and iterative non-linear regression algorithms known as Newton–Gauss–Levenberg/Marquardt (NGL/M) [15]. Herein, in the NGL/M algorithm, the non-linear parameters subjected to fitting were the *K_m_* (metabolism constant), *υ_max_* (maximum velocity), and C_0_ (initial concentration of an involved component) of the reaction mechanism defining the matrix of metabolite profiles. These parameters were refined to minimize the sum of the squares of the residual matrix. The non-linearity of the parameters arises from the inherent non-linear nature of the relationship between the parameters and the residuals.

In the iterative NGL/M algorithm, the process begins with an initial estimation of each parameter (p). The parameter vector is then refined by incorporating an appropriate parameter shift vector δp, leading to a more refined fit, although it may still not be perfect. This iterative process is repeated until the optimal solution is achieved [16].

The parameters of the model were fitted based on measured data by minimizing the sum of the mean squared error (SMSE) between the measured and estimated model output as follows:

(1) $$SMSE=\frac{\sum_{i}^{n}{\left(y_{i}-{ŷ}_{i}\right)}^{2}}{n}$$

where *n* is the number of data points, *y_i_* is the experimental measured data, and ŷ_i_ is the corresponding predicted result.

The concentration profiles of all the involved species were modeled using a set of numerical ordinary differential equations (ODE solver) that describe the hard model of the corresponding biokinetic equations. Michaelis–Menten kinetics was employed to investigate the kinetic parameters and optimize the *K_m_* and *υ_max_* values.

### 2.2. Global Sensitivity Analysis

Sensitivity analysis (SA) is a critical step in identifying and prioritizing significant reactions and parameters, as well as examining how variations in input parameters impact the output of a system or model. This analysis facilitates a comprehensive understanding of the system’s sensitivity to changes in each input parameter. Its applicability extends across diverse fields, including engineering, environmental science, systems control, process optimization, chemical and reactor design, and cell biology.

These valuable mathematical tools hold significant importance in modeling and the assessment of risks associated with complex systems, categorically falling into three distinct types: screening methods, local sensitivity analysis methods, and global sensitivity analysis methods. Conventional local sensitivity methods typically rely on linear assumptions and encounter limitations when applied to nonlinear systems. In contrast, the more complicated global sensitivity methods may pose challenges due to time constraints.

Global sensitivity analysis (GSA) offers valuable insights into the robustness and reliability of a model or system, shedding light on the impact of design variables on its performance. Such information is valuable for informing optimization processes. The input parameters within a system encompass a range of factors, including physicochemical attributes, such as kinetic constants and thermodynamic equilibria, along with initial conditions and operating conditions. As physicochemical parameters are commonly derived from experimental measurements or theoretical calculations, inherent uncertainties often accompany these values. So, GSA played a crucial role in categorizing parameters according to their impact reduction. Furthermore, it facilitated the exclusion of parameters that had a minimal influence on the model sensitivity from the subsequent optimization cycle, thus maintaining them at their initial values [15].

Various types of global sensitivity analyses are available, including the weighted average of local sensitivity analysis, partial rank correlation coefficient, multiparametric sensitivity analysis, Fourier amplitude sensitivity analysis, and Sobol’s method. Among these methods, Sobol sensitivity analysis based on variance decomposition stands out as one of the most powerful techniques thus far [17].

Sobol introduced the variance-based method, along with its corresponding sensitivity concept, which relies on a high-dimensional model representation. This method breaks down the variance of the model output Y = *f*(***X***) = *f* (*X*_1_, *X*_2_, *…*, *X_n_*) into different terms (first order, second order, and higher orders), accounting for the input parameters and their interactions, as shown in the following multidimensional expression:

(2) $$Y=f\left(X\right)=f_{0}+\sum_{i=1}^{n}f_{i}\left(X_{i}\right)+\sum_{i=1}^{n}\sum_{j>i}^{n}f_{i,j}\left(X_{i},X_{j}\right)+\ldots +f_{1,2\ldots n}(X_{1},X_{2},\ldots X_{n})\;$$

where *f*_0_, *f_i_* (*X_i_*), and *f_ij_* (*X_i_*, *X_j_*) represent the mean, first-order terms, and second-order terms of *f*(*X*), respectively, and *X_i_* ϵ [0, 1]; i = 1, 2, …, *n*.

Several types of Sobol indices exist. However, calculating higher orders becomes unfeasible with a large number of input variables. The first-order sensitivity index (S_i_) quantifies the extent to which each uncertain parameter contributes to the variance in the model output:

(3) $$S_{i}=\frac{V[E[Y\;⎸X_{i}]]}{V[Y]}$$

Here, 𝔼 [*Y*⎸*X_i_*] represents the expected value of the output *Y* when parameter *X_i_* is fixed and 𝕍 [*Y*] is the total variance of the response. A low sensitivity index for a parameter indicates that variations in that parameter result in relatively minor changes in the final model output. Conversely, a high sensitivity index for a parameter suggests that alterations to it cause significant shifts in the model output. Each *S_i_* informs us about the anticipated reduction in the model’s variance when parameter *X_i_* is held constant. It should be noted that the total of the first-order Sobol sensitivity indices cannot exceed one.

## 3. Methodology

### 3.1. The Mathematical Model

Mathematical models offer substantial potential for streamlining experimental processes, leading to cost savings and reduced time requirements for labor-intensive laboratory investigations. Additionally, they contribute to a deeper comprehension of the underlying mechanisms governing these processes. Metabolic flux analysis (MFA) has found extensive application in biotechnology for characterizing the state of cellular metabolism. This analysis relies on pseudo-steady-state mass balances around intracellular metabolites. The metabolic flux rate is determined by the substrate concentrations using Michaelis–Menten-type kinetics. The flux kinetic equation is formulated as follows:

(4) $$\upsilon =\frac{\upsilon_{max}\;.\;[S]}{K_{m}+[S]}$$

Here, υ denotes the reaction rate or flux, *υ_max_* represents the maximum reaction rate (attained when the enzyme is saturated with the substrate), [*S*] denotes the substrate concentration, and *K_m_* is the Michaelis–Menten affinity constant. *K_m_* signifies the substrate concentration at which the reaction rate is half of *υ_max_*.

### 3.2. Kinetic Model of the Bioprocesses

The primary aim of this research was to construct a model that accurately characterized the dynamics of AAV production within a bioreactor culture. In the current phase of model development, the HEK293 cell was treated as a single compartment, with no consideration given to intracellular sub-compartments, such as the mitochondria or the nucleus. The dynamic model comprised 32 reactions, incorporating a total of 89 kinetic parameters (*K_m_* = 52, *υ_max_* = 37). The network employed in this study is outlined in Table 1, which encompasses key metabolic pathways, namely, the glycolysis, tricarboxylic acid cycle (TCA), pyruvate fates, pentose phosphate pathway, anaplerotic reaction, amino acid metabolism, nucleotides synthesis, biomass synthesis, and the metabolic pathways of protein synthesis of the AAV (capsid and Rep proteins). The sequences of recombinant proteins were sourced from the National Institutes of Health (NIH). A total of fifty-three ordinary differential equations, which represented mass balances in the model, were considered.

## 4. Experimental Data Sets

**Figure 1:**
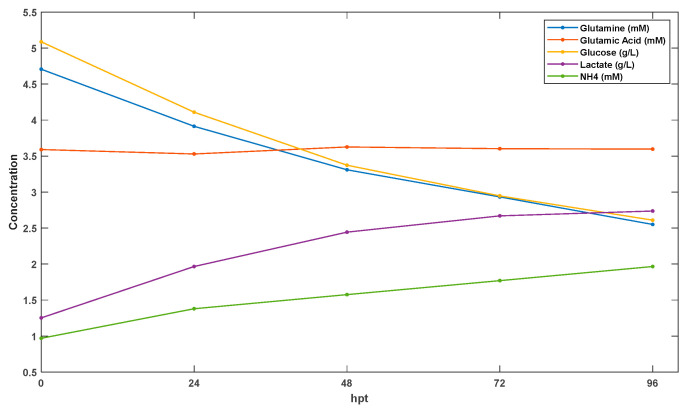
Metabolite profiles of production of adeno-associated virus 6 (AAV6) via triple transient transfection in low-cell-density culture, including concentration profiles of glutamine (mM), glutamic acid (mM), glucose (g/L), lactate (g/L), and ammonium (mM).

In this study, we utilized a previously published dataset [18] to develop a metabolic model framework based on flux kinetics related to AAV production in the HEK293 cell line platform. This dataset includes metabolite profiles observed during the production of AAV6 in TubeSpin bioreactors under a low-cell-density culture (LCD: 1 × 10^6^ cells mL^−1^). At 24 h intervals, samples of the culture were collected and then subjected to centrifugation to separate the cellular components. The supernatant obtained after centrifugation was analyzed to quantify the levels of key nutrients and metabolites, including glutamine, glutamic acid, glucose, lactate, and ammonium. The data utilized in this study are presented in Figure 1. The initial concentration at time zero was known for these five species, while the fitting process required knowledge of the initial concentration for all the species. However, since such information was only available for five of the species, adjustments to the initial concentrations of the remaining species, as well as the model parameters, were made during the optimization iterations. Further details about this real-world example can be found in another source [18].

## 5. Results and Discussion

To optimize the AAV production, a suitable mathematical representation of the process was indispensable. The inherent complexity of even the simplest living cell means that any mathematical depiction serves as a rough approximation. Indeed, a mathematical model is inherently a simplification of system behavior. In the modeling realm, it is an accepted theory that a single set of data can be compatible with multiple models. In this study, adhering to Occam’s razor theory and acknowledging the limited available data, as well as the absence of initial information (such as the initial concentrations of all the involved components), we opted for a simpler model for the optimization of AAV production via HEK293 cells.

### 5.1. Model Optimization

To compute the parameters of the developed model, we employed the NGL/M parameter estimation procedure. The initial parameter estimates were derived from the literature. Given the large number of parameters in the model (142), we optimized them by fitting 2–10 parameters at a time while keeping the rest constant. This process was iteratively repeated, with the fitted parameters replaced by another group in each iteration. We continued this cycle until the fitted parameters no longer changed significantly, and the sum of the squared residuals reached its minimum value. The optimized kinetic parameters (*K_m_*, *υ_max_*), in addition to the initial composition and the concentrations (*C_0_*) in the bioreactor medium, are listed in Table 2 and Table 3 respectively.

**Figure 2:**
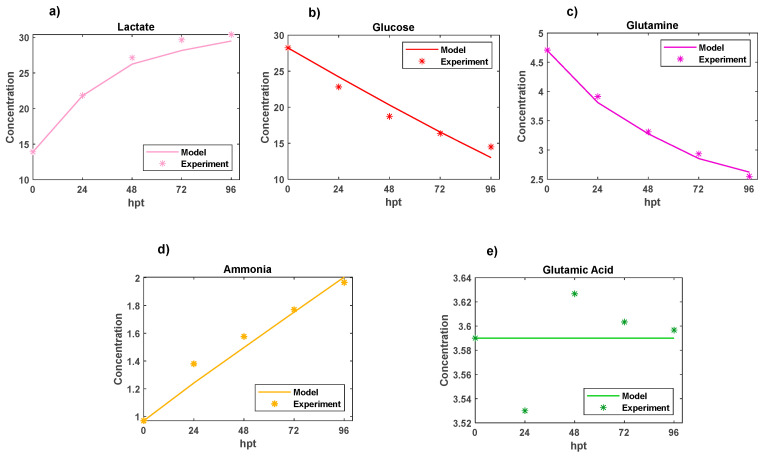
Comparison of experimental (star markers) and recovered (line) kinetic profiles of five metabolites: (**a**) lactate (mM), (**b**) glucose (mM), (**c**) glutamine (mM), (**d**) ammonium (mM), and (**e**) glutamic acid (mM).

In Figure 2, the calculated kinetic metabolite profiles are represented by lines, with experimentally measured data points indicated by star markers. The profiles obtained were well recovered, and the calculated and measured profiles generally exhibited good agreement, with the exception of the glutamic acid profile. The observed lack of a good fit was attributed to the absence of enough information regarding the profile of glutamic acid in the model. Specifically, this species has solely been considered in the construction of AAV proteins (Rep and Cap) within the model.

The total sum of the squares of residuals, denoting the differences between the real and modeled profiles, was 7.07 mM, with individual contributions as follows: ssq-_Ammonia_ = 0.027 mM, ssq-_Lactate_ = 0.268 mM, ssq-_Glucose_ = 6.746 mM, ssq-_Glutamic Acid_ = 0.005 mM, and ssq-_Glutamine_ = 0.022 mM. The greatest ssq was associated with glucose, a result of its higher profile intensity compared with the other metabolites. Indeed, the higher intensity translated to a more significant impact on the sum of the squares of residuals (ssq), which contributed to a notable difference in the deviation.

### 5.2. Sensitivity Analysis

Given the complexity of the system, the model yielded a considerable number of parameters. Consequently, a Sobol-based global sensitivity analysis (SGSA) of the kinetic parameters was carried out to assess the model’s dependence on specific parameters over time. Considering that model inputs can encompass a wide range, including parameters *K_m_* and *υ_max_*, global sensitivity analysis has emerged as an innovative approach for identifying which reactions and processes have the greatest significant impact on the behavior of the overall system.

For this, all parameters were simultaneously varied across the entire parameter space. Each parameter was defined to cover a specific finite range (specify the range including the lower and upper bounds for each), distributed uniformly. Then, a series of model outputs utilized the defined ODE model, which covered various ranges of parameter uncertainties, was generated. The model output was a random variable with a mean (*f*_0_) and total variance (*𝕍*). Finally, the Sobol method was employed to decompose *𝕍* into contributions from individual parameter effects (Equation (3)).

**Figure 3:**
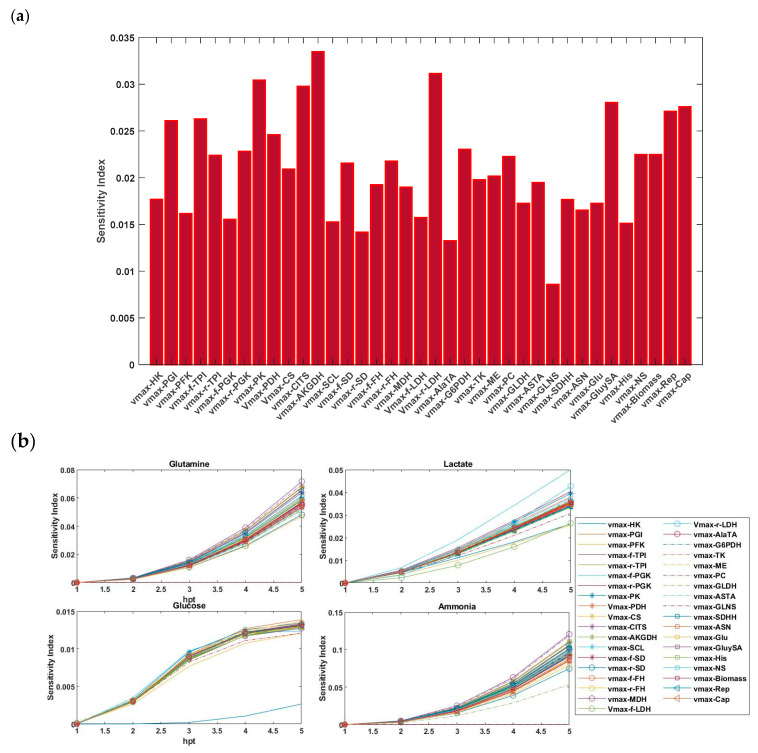
(**a**) First-order Sobol sensitivity indices of the 37 *V_max_* parameters. (**b**) Changes in the sensitivity indices concerning the glutamine, glucose, ammonia, and lactate output concentrations over time.

**Figure 4:**
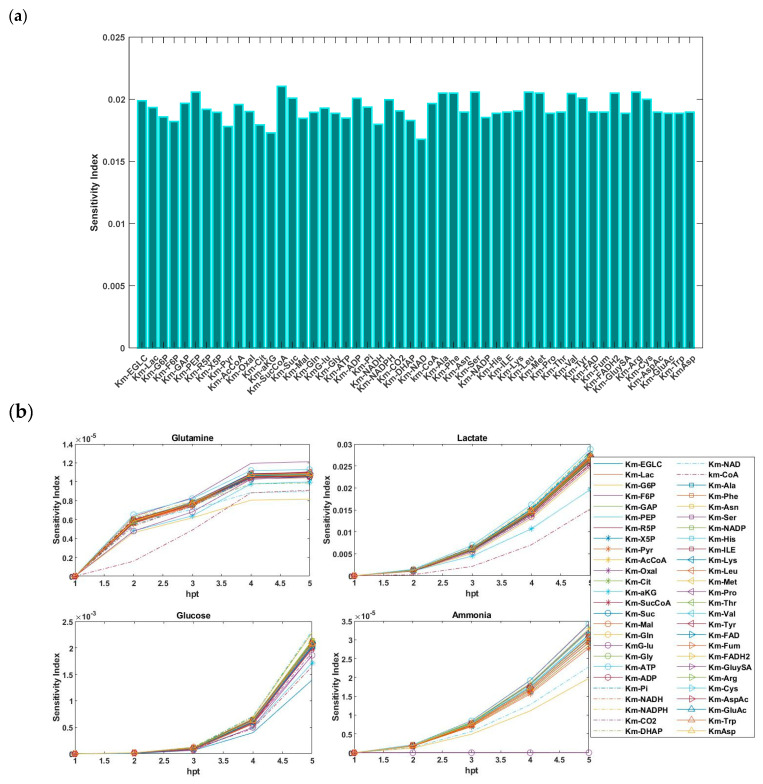
(**a**) First-order Sobol sensitivity indices of the 52 *K_m_* parameters. (**b**) Changes in the sensitivity indices concerning the glutamine, glucose, ammonia, and lactate output concentrations over time.

For the SGSA, we explored two alternative perspectives. First, the sensitivity results of 37 *v_max_* and 52 *K_m_* parameters of the system were computed and are presented in Figure 3a and Figure 4a, respectively. In Figure 3a, the results indicate that parameters *v_max-PGI_*, *v_max-f-TPI_*, *v_max-PK_*, *v_max-CITS_*, *v_max-AKGDH_*, *v_max-r-LDH_*, *v_max-GluySA_*, *v_max-Rep_*, and *v_max-Cap_* were the primary contributors, where they explained approximately 26% of the variability in the model output. This was followed by the significant parameters *v_max-r-TPI_*, *v_max-r-PGK_*, *v_max-PDH_*, *v_max-CS_*, *v_max-f-SD_*, *v_max-r-FH_*, *v_max-G6PDH_*, *v_max-PC_*, *v_max-NS_*, and *v_max-Biomass_*, which collectively contributed an additional 22% of the variation. However, as seen in Figure 4a, the magnitudes of the sensitivity indices for all *K_m_* parameters were slightly varied but remained approximately close to each other.

Next, we investigated the relative influence of the uncertainty in these parameters (*v_max_*, *K_m_*) on the output concentrations of glutamine, glucose, lactate, and ammonium. Indeed, the sensitivity index reflected the influence of each parameter on the output at the specified time. For each profile concentration output, the importance of the parameters was ranked (Figure 3b and Figure 4b). Due to the absence of enough information regarding the profile of glutamic acid in the model, this component was neglected. It was observed that the sensitivity indices, and consequently, the degree of importance of the parameters, varied for each profile.

Our study provides an initial investigation into metabolic pathways associated with rAAV vector production. Using a simplified model, we identified pathways that could inform future studies. Given the inherent complexity and variability of biological systems, fully comprehensive models remain challenging to develop. However, simplified models can still offer valuable insights by approximating system behaviour [19].

We recognize that the proposed model, constrained by the available dataset, does not fully capture the complexity of rAAV production. However, the extracted parameters provide useful insights and indicate a degree of predictive potential for rAAV yields. While not comprehensive, this model offers a foundation for refining future models and informing metabolic engineering strategies to address challenges in large-scale rAAV manufacturing.

## 6. Conclusions

In this paper, we propose a metabolic behavior model for AAV production in an HEK293 cell line culture. The model, describing metabolic network flux kinetics, was calibrated to estimate key parameters. We utilized a measured dataset that contained five metabolite profiles from the production of adeno-associated virus 6 via triple transient transfection in a low-cell-density culture. These profiles included concentrations of glutamine (mM), glutamic acid, glucose, lactate, and ammonium. The model was applied to the experimental dataset, which allowed for the extraction of key parameters. Additionally, a Sobol-based global sensitivity analysis was conducted to identify and assess the most sensitive parameters of the model. As expected in such a complex system, multiple critical parameters exhibited interdependencies and varied with external conditions.

While this approach provides a structured framework for studying metabolic behaviors in AAV production, the findings should be interpreted with caution due to the limited dataset used for the model calibration. Expanding the dataset with more diverse conditions and additional validation experiments would be necessary to further assess the model’s reliability and general applicability. Despite these limitations, the model offers preliminary insights into key metabolic processes in HEK293 cells, which could support future efforts to optimize AAV production.

## 7. Limitations and Future Work

While incorporating additional datasets would provide a more comprehensive understanding of metabolic pathways in rAAV vector production, the present study was limited by constraints in experimental facilities and sample collection. As a result, the findings should be interpreted within the context of the specific dataset used.

To improve the model’s applicability, further optimization of different datasets is necessary. Although the model describes metabolic behaviors within the given dataset, its parameters may not be directly transferable to other conditions without recalibration. Variations in culture conditions, measurement techniques, and experimental setups could impact model predictions, requiring additional validation.

Given these limitations, the model should be considered an initial step toward understanding metabolic dynamics in rAAV production rather than a definitive predictive tool. Future work will focus on expanding the dataset, refining the parameter estimation, and validating the model across different production environments to enhance its robustness and generalizability.

## Figures and Tables

**Table 1:** Reactions of the metabolic network of the AAV production via an HEK293 cell.

#	Metabolic Pathway	Metabolic Reaction Network
υ_1_	Glycolysis	Glc + ATP → G_6_P + ADP
υ_2_		G_6_P → F_6_P
υ_3_		F_6_P + ATP → DHAP + G_3_P + ADP
υ_4_		DHAP ↔ G_3_P
υ_5_		G_3_P + NAD^+^ + ADP ↔ PEP + NADH + ATP
υ_6_		PEP + ADP → Pyr + ATP
υ_7_	Tricarboxylic acid cycle	Pyr + NAD^+^ + CoA → AcCoA + CO_2_ + NADH
υ_8_		AcCoA + Oxal + H_2_O → Cit + CoA
υ_9_		Cit + NADP^+^ → αKG + CO_2_ + NADPH
υ_10_		αKG + CoA + NAD^+^ → SucCoA + CO_2_ + NADH
υ_11_		SucCoA + ADP + Pi → Suc + ATP + CoA
υ_12_		Suc + FAD ↔ Fum + FADH_2_
υ_13_		Fum ↔ Mal
υ_14_		Mal + NAD^+^ → Oxal + NADH
υ_15_	Pyruvate fates	G_6_P + 2NADP^+^ + H_2_O → R_5_P + 2NADPH + CO_2_
υ_16_		2X5P + R_5_P → 2F_6_P + G_3_P
υ_17_	Anaplerotic reaction	Mal + NADP^+^ → Pyr + CO_2_ + NADPH
υ_18_		Pyr + CO_2_ + ATP → Oxal
υ_19_	Amino acid metabolism	Glu + NAD^+^ → αKG + NH_4_ + NADH
υ_20_		Oxal + Glu → Asp + αKG
υ_21_		Gln → Glu + NH_4_
υ_22_		Ser → Pyr + NH_4_
υ_23_		Asn → Asp + NH_4_
υ_24_		Glu + ATP + 2NADPH + H^+^ → Pro^-^ + ADP + 2NADP^+^
υ_25_		GluySA → Pro^-^
υ_26_		His → Glu + NH_4_
υ_27_	Nucleotides synthesis	2Asp + Gly + 2Gln + 0.6R_5_P + CO_2_ → 2Mal + 2Glu
υ_28_	Pentose phosphate pathway	G_6_P + 2NADP^+^ + H_2_O → R_5_P + 2NADPH + CO_2_
υ_29_		2X5P + R_5_P → 2F_6_P + G_3_P
υ_30_	Biomass synthesis	0.024*R5P + 0.029*G6P + 0.04*Gln + 0.013*Ala + 0.007*Arg + 0.026*Asp + 0.003*His + 0.0084*Ile + 0.013*Leu + 0.01*Lys + 0.099*Ser + 0.004*Tyr + 0.0096*Val + 0.016*Gly + 3.78*ATP → Biomass
υ_31_	Protein folding	0.05*Ala + 0.03*Cys + 0.06*AspAc + 0.07*GluAc + 0.03*Phe + 0.05*Gly + 0.02*His + 0.06*ILE + 0.08*Lys + 0.08*Leu + 0.02*Met + 0.04*Asn + 0.05*Pro + 0.05*Glu + 0.04*Arg + 0.07*Ser + 0.07*Thr + 0.06*Val + 0.03*Trp + 0.03*Tyr + ATP → Rep + ADP
υ_32_		0.07*Ala + 0.01*Cys + 0.06*AspAc + 0.05*GluAc + 0.05*Phe + 0.08*Gly + 0.02* His + 0.03* ILE + 0.05* Lys + 0.06* Leu + 0.01*Met + 0.09*Asn + 0.08*Pro + 0.05*Glu + 0.04*Arg + 0.07*Ser + 0.07*Thr + 0.05*Val + 0.02*Trp + 0.04*Tyr + ATP → Cap + ADP

**Table 2:** The equations of the metabolite fluxes of the considered model and their fitted parameters *K_m_* and *υ_max_*.

Flux	Equations	Parameters
υ_HK_	$\upsilon_{HK}=\upsilon_{maxHK}\frac{Glc}{K_{mGlc}+Glc}\frac{ATP}{K_{mATP}+ATP}$	*υ_maxHK_* = 0.205 *K_mGlc_*_=_ 5.100 *K_mATP_* = 0.594
υ_PGI_	$\upsilon_{PGI}=\upsilon_{maxPGI}\frac{G_{6}P}{K_{mG_{6}P}+G_{6}P}$	*υ_maxPGI_* = 2.283 *K_mG6P_* = 2.165
υ_PFK_	$\upsilon_{PFK}=\upsilon_{maxPFK}\frac{F_{6}P}{K_{mF6P}+F_{6}P}\frac{ATP}{K_{mATP}+ATP}$	*υ_maxPFK_* = 6.667 *K_mF6P_* = 0.016
υ_TPI_	$\upsilon_{TPI}=\upsilon_{max-f-TPI}\frac{DHAP}{K_{mDHAP}+DHAP}-\upsilon_{max-r-TPI}\frac{GAP}{K_{mGAP}+GAP}$	*υ_max-f-TPI_* = 1.296 *K_mDHAP_* = 0.043 *υ_max-r-TPI_* = 14.299 *K_mGAP_* = 4.6 × 10^−6^
υ_PGK_	$\upsilon_{PGK}=\upsilon_{max-f-PGK}\frac{GAP}{K_{mGAP}+GAP}\frac{NAD}{K_{mNAD}+NAD}\frac{ADP}{K_{mADP}+ADP}\;-\upsilon_{max-r-PGK}\frac{PEP}{K_{mPEP}+PEP}\frac{NADH}{K_{mNADH}+NADH}\frac{ATP}{K_{mATP}+ATP}$	*υ_max-f-PGK_* = 3.841 *υ_max-r-PGK_* = 4.0 × 10^−3^ *K_mNAD_* = 2.4 × 10^−5^ *K_mADP_* = 4.3 × 10^−7^ *K_mPEP_* = 0.018 *K_mNADH_* = 0.017
υ_PK_	$\upsilon_{PK}=\upsilon_{maxPK}\frac{PEP}{K_{mPEP}+PEP}\frac{ADP}{K_{mADP}+ADP}$	*υ_maxPK_* = 2.520
υ_PDH_	$\upsilon_{PDH}=\upsilon_{maxPDH}\frac{Pyr}{K_{mPYr}+Pyr}\frac{NAD}{K_{mNAD}+NAD}\frac{CoA}{K_{mCoA}+CoA}$	*υ_maxPDH_* = 0.571 *K_mPY_r* = 0.013 *K_mCoA_* = 6.4 × 10^−4^
υ_CS_	$\upsilon_{CS}=\upsilon_{maxCS}\frac{AcCoA}{K_{mAcCoA}+AcCoA}\frac{Oxal}{K_{mOxal}+Oxal}$	*υ_maxCS_* = 0.125 *K_mAcCoA_* = 0.012 *K_mOxal_* = 0.004
υ_CITS_	$\upsilon_{CITS}=\upsilon_{maxCITS}\frac{Cit}{K_{mCit}+Cit}\frac{NADP}{K_{mNADP}+NADP}$	*υ_maxCITS_* = 1.634 *K_mCit_* = 0.082 *K_mNADP_* = 6.9 × 10^−4^
υ_AKGDH_	$\upsilon_{AKGDH}=\upsilon_{maxAKGDH}\frac{aKG}{K_{maKG}+aKG}\frac{CoA}{K_{mCoA}+CoA}\frac{NAD}{K_{mNAD}+NAD}$	*υ_maxAKGDH_* = 1.496 *K_maKG_* = 1.9 × 10^−5^
υ_SCL_	$\upsilon_{SCL}=\upsilon_{maxSCL}\frac{SucCoA}{K_{mSucCoA}+SucCoA}\frac{ADP}{K_{mADP}+ADP}\frac{Pi}{K_{mPi}+Pi}$	*υ_maxSCL_* = 1.300 *K_mSucCoA_* = 6 × 10^−4^ *K_mPi_* = 0.047
υ_SD_	$\upsilon_{SD}=\upsilon_{max-f-SD}\frac{Suc}{K_{mSuc}+Suc}\frac{FAD}{K_{mFAD}+FAD}\;-\upsilon_{max-r-SD}\frac{FUM}{K_{mFUM}+FUM}\frac{{FADH}_{2}}{K_{mFADH2}+{FADH}_{2}}$	*υ_max-f-SD_* = 2.378 *υ_max-r-SD_* = 1.989 *K_mSuc_* = 0.034 *K_mFAD_* = 1.320 *K_mFUM_* = 0.041 *K_mFADH2_* = 0.053
υ_FH_	$\upsilon_{FH}=\upsilon_{max-f-FH}\frac{Fum}{K_{mFum}+Fum}-\upsilon_{max-r-FH}\frac{Mal}{K_{mMal}+Mal}$	*υ_max-f-FH_* = 8.3 × 10^−2^ *υ_max-r-FH_* = 1.777 *K_mMal_* = 0.046
υ_MDH_	$\upsilon_{MDH}=\upsilon_{maxMDH}\frac{Mal}{K_{mMal}+Mal}\frac{NAD}{K_{mNAD}+NAD}$	*υ_maxMDH_* = 1.4 × 10^−2^
υ_LDH_	$\upsilon_{LDH}=\upsilon_{max-f-LDH}\frac{Pyr}{K_{mPyr}+Pyr}\frac{NADH}{K_{mNADH}+NADH}\;-\upsilon_{max-r-LDH}\frac{Lac}{K_{mLac}+Lac}\frac{NAD}{K_{mNAD}+NAD}$	*υ_max-f-LDH_* = 0.565 *υ^max-r-LDH^* = 0.275 *K_mLac_* = 3.119
υ_AlaTA_	$\upsilon_{AlaTA}=\upsilon_{maxAlaTA}\frac{Pyr}{K_{mPyr}+Pyr}\frac{Glu}{K_{mGlu}+Glu}$	*υ_maxAlaTA_* = 3.1 × 10^−2^ *K_mGlu_* = 1.7 × 10^−4^
υ_G6PDH_	$\upsilon_{G6PDH}=\upsilon_{maxG6PDH}\frac{G_{6}P}{K_{mG_{6}P}+G_{6}P}\frac{NADP}{K_{mNADP}+NADP}$	*υ_maxG6PDH_* = 5 × 10^−3^
υ_TK_	$\upsilon_{TK}=\upsilon_{maxTK}\frac{X_{5}P}{K_{mX_{5}P}+X_{5}P}\frac{R_{5}P}{K_{mR5P}+R_{5}P}$	*υ_maxTK_* = 4 × 10^−3^ *K_mX5P_* = 7 × 10^−3^ *K_mR5P_* = 6.5 × 10^−2^
υ_ME_	$\upsilon_{ME}=\upsilon_{maxME}\frac{Mal}{K_{mMal}+Mal}\frac{NADP}{K_{mNADP}+NADP}$	*υ_maxME_* = 0.84
υ_PC_	$\upsilon_{PC}=\upsilon_{maxPC}\frac{Pyr}{K_{mPyr}+Pyr}\frac{CO_{2}}{K_{mCO2}+CO_{2}}\frac{ATP}{K_{mATP}+ATP}$	*υ_maxPC_* = 9.02 *K_mCO2_* = 0.45
υ_GLDH_	$\upsilon_{GLDH}=\upsilon_{maxGLDH}\frac{Glu}{K_{mGlu}+Glu}\frac{NAD}{K_{mNAD}+NAD}$	*υ_maxGLDH_* = 2.3 × 10^−2^
υ_ASTA_	$\upsilon_{ASTA}=\upsilon_{maxASTA}\frac{Oxal}{K_{mOxal}+Oxal}\frac{Glu}{K_{mGlu}+Glu}$	*υ_maxASTA_* = 1.21
υ_GLNS_	$\upsilon_{GLNS}=\upsilon_{maxGLNS}\frac{Gln}{K_{mGln}+Gln}$	*υ_maxGLNS_* = 0.01
υ_SDHH_	$\upsilon_{SDHH}=\upsilon_{maxSDHH}\frac{Ser}{K_{mSer}+Ser}$	*υ_maxSDHH_* = 0.43 *K_mSer_* = 0.96
υ_ASN_	$\upsilon_{ASN}=\upsilon_{maxASN}\frac{Asn}{K_{mAsn}+Asn}$	*υ_maxASN_* = 1 × 10^−4^ *K_mAsn_* = 1.7 × 10^−3^
υ_Glu_	$\upsilon_{Glu}=\upsilon_{maxGlu}\frac{Glu}{K_{mGlu}+Glu}\frac{ATP}{K_{mATP}+ATP}\frac{NADPH}{K_{mNADPH}+NADPH}$	*υ_maxGlu_* = 0.98 *K_mNADPH_* = 8.06 × 10^−5^
υ_GluySA_	$\upsilon_{GluySA}=\upsilon_{maxGluySA}\frac{GluySA}{K_{mGluySA}+GluySA}$	*υ_maxGluySA_* = 8.5 × 10^−2^ *K_mGluySA_* = 2.15 × 10^−3^
υ_His_	$\upsilon_{His}=\upsilon_{maxHis}\frac{His}{K_{mHis}+His}$	*υ_maxHis_* = 6 × 10^−4^ *K_mHis_* = 1.89 × 10^−2^
υ_NS_	$\upsilon_{NS}=\upsilon_{maxNS}\frac{Asp}{K_{mAsp}+Asp}\frac{Gly}{K_{mGly}+Gly}\frac{Gln}{K_{mGln}+Gln}\frac{R_{5}P}{K_{mR5P}+{R5}_{P}}\frac{CO_{2}}{K_{mCO2}+{CO}_{2}}$	*υ_maxNS_* = 0.79 *K_mAsp_* = 1.29 × 10^−5^ *K_mGly_* = 1.51 × 10^−5^ *K_mGln_* = 3 × 10^−4^
υ_Growth_	$\upsilon_{Growth}=\upsilon_{maxGrowth}\frac{R_{5}P}{K_{mR5P}+{R5}_{P}}\frac{G_{6}P}{K_{mG6P}+G_{6}P}\frac{Gln}{K_{mGln}+Gln}\frac{Ala}{K_{mAla}+Ala}$ $\frac{Arg}{K_{mArg}+Arg}\frac{Asp}{K_{mAsp}+Asp}\frac{His}{K_{mHis}+His}\frac{Ile}{K_{mIle}+Ile}\frac{Leu}{K_{mLeu}+Leu}\frac{Lys}{K_{mLys}+Lys}$ $\frac{Ser}{K_{mSer}+Ser}\frac{Tyr}{K_{mTyr}+Tyr}\frac{Val}{K_{mVal}+Val}\frac{Gly}{K_{mGly}+Gly}\frac{ATP}{K_{mATP}+ATP}$	*υ_Growth_* = 1.8 × 10^−2^ *K_mAla_* = 2.8 × 10^−2^ *K_mArg_* = 1.2 × 10^−2^ *K_mIle_* = 1.7 × 10^−2^ *K_mLeu_* = 3.9 × 10^−4^ *K_mLys_* = 5.8 × 10^−2^ *K_mTyr_* = 8.1 × 10^−3^ *K_mVal_* = 0.60
υ_Rep_	$\upsilon_{Rep}=\upsilon_{maxRep}\frac{Ala}{K_{mAla}+Ala}\frac{Cys}{K_{mcys}+Cys}\frac{AspAc}{K_{mAspAc}+AspAc}\frac{GluAc}{K_{mGluAc}+GluAc}$ $\frac{Phe}{K_{mPhe}+Phe}\frac{Gly}{K_{mGly}+Gly}\frac{His}{K_{mHis}+His}\frac{Ile}{K_{mIle}+Ile}\frac{Lys}{K_{mLys}+Lys}\frac{Leu}{K_{mLeu}+Leu}$ $\frac{Met}{K_{mMet}+Met}\frac{Asn}{K_{mAsn}+Asn}\frac{Pro}{K_{mPro}+Pro}\frac{Glu}{K_{mGlu}+Glu}\frac{Arg}{K_{mArg}+Arg}\frac{Ser}{K_{mSer}+Ser}$ $\frac{Thr}{K_{mThr}+Thr}\frac{Val}{K_{mVal}+Val}\frac{Trp}{K_{mTrp}+Trp}\frac{Tyr}{K_{mTyr}+Tyr}\frac{ATP}{K_{mATP}+ATP}$	*υ_maxRep_* = 2.5 × 10^−3^ *K_mCys_* = 4.94 × 10^−2^ *K_mAspAc_* = 4.95 × 10^−2^ *K_mGluAc_* = 5.03 × 10^−2^ *K_mPhe_* = 7.1 × 10^−2^ *K_mMet_* = 0.32 *K_mPro_* = 4.74 × 10^−2^ *K_mThr_* = 1.94 × 10^−2^ *K_mTrp_* = 4.99 × 10^−2^
υC_ep_	$\upsilon_{Cep}=\upsilon_{maxCep}\frac{Ala}{K_{mAla}+Ala}\frac{Cys}{K_{mSys}+Cys}\frac{AspAc}{K_{mAspAc}+AspAc}\frac{GluAc}{K_{mGluAc}+GluAc}$ $\frac{Phe}{K_{mPhe}+Phe}\frac{Gly}{K_{mGly}+Gly}\frac{His}{K_{mHis}+His}\frac{Ile}{K_{mIle}+Ile}\frac{Lys}{K_{mLys}+Lys}\frac{Leu}{K_{mLeu}+Leu}$ $\frac{Met}{K_{mMet}+Met}\frac{Asn}{K_{mAsn}+Asn}\frac{Pro}{K_{mPro}+Pro}\frac{Glu}{K_{mGlu}+Glu}\frac{Arg}{K_{mArg}+Arg}\frac{Ser}{K_{mSer}+Ser}$ $\frac{Thr}{K_{mThr}+Thr}\frac{Val}{K_{mVal}+Val}\frac{Trp}{K_{mTrp}+Trp}\frac{Tyr}{K_{mTyr}+Tyr}\frac{ATP}{K_{mATP}+ATP}$	*υ_maxCep_* = 2.9 × 10^−2^

**Table 3:** The fitted initial concentrations of all the involved species.

Metabolite	Name	Fitted Value	Metabolite	Name	Fitted Value
ATP	ATP	19.95 mM	His	Histidine	0.604 mM
ADP	ADP	0.175 mM	Ile	Isoleucine	0.777 mM
AcCoA	Acetyl-CoA	0.016 mM	Lac ^1^	Lactate	13.91 mM
αKG	Alpha-ketoglutarate	0.110 mM	Lys	Lysine	0.017 mM
Ala	Alanine	6.985 mM	Leu	Leucine	0.517 mM
Asp	Aspartate	3.065 mM	Mal	Malate	4.262 mM
Asn	Asparagine	0.495 mM	Met	Methionine	1.150 mM
Arg	Arginine	0.519 mM	NAD NADH	Nicotinamide adenine dinucleotide	0.634 mM 0.029 mM
AspAc	Aspartic acid	0.011 mM	NADP NADPH	Nicotinamide adenine dinucleotide phosphate	0.009 mM 0.106 mM
CoA	Coenzyme A	1.132 mM	NH_4_^1^	Ammonia	0.97 mM
Cit	Citrate	0.810 mM	Oxal	Oxaloacetate	0.003 mM
CO_2_	CO_2_	3.530 mM	Pep	3-phosphoglycerate	0.309 mM
Cys	Cysteine	0.248 mM	Pyr	Pyruvate	7.291 mM
DHAP	dihydroxyacetone phosphate	3.072 mM	Pi	Orthophosphate or hydrogenphosphate	0.498 mM
F_6_P	Fructose-6-phosphate	0.685 mM	Pro	Proline	1.520 mM
FAD	flavin adenine dinucleotide	3.630 mM	Phe	Phenylalanine	0.874 mM
Fum	Fumaric acid	0.192 mM	R_5_P	Ribulose-5-phosphate	1.169 mM
FADH_2_		0.753 mM	SucCoA	Succinate Coenzyme A	0.007 mM
GLC ^1^	Glucose	28.23 mM	Suc	Succinate	0.997 mM
GAP	Glyceraldehyde 3-phosphate	0.600 mM	Ser	Serine	0.012 mM
G_6_P	Glucose-6-phosphate	1.255 mM	Thr	Threonine	0.205 mM
Glu	Glutamate	2.82 × 10^−5^ mM	Trp	Tryptophan	0.237 mM
Gln ^1^	Glutamine	4.7 mM	Tyr	Tyrosine	0.255 mM
GluySA	Glutamateγ−semialdehyde	0.034 mM	Val	Valine	0.107 mM
GluAc ^1^	Glutamic acid	3.59 mM	X_5_P	Xylulose 5 − phosphate	0.004 mM
Gly	Glycine	1.155 mM			

^1^ The initial concentrations of these species were included in the available data.

## Data Availability

The data that support the findings of this study are available from the corresponding author upon reasonable request.
